# Computational Study of Chalcogenide-Based Perovskite Solar Cell Using SCAPS-1D Numerical Simulator

**DOI:** 10.3390/ma18010186

**Published:** 2025-01-04

**Authors:** Edson L. Meyer, Sinikiwe A. Mvokwe, Opeoluwa O. Oyedeji, Nicholas Rono, Mojeed A. Agoro

**Affiliations:** 1 Fort Hare Institute of Technology, University of Fort Hare, Private Bag X1314, Alice 5700, Eastern Cape, South Africamagoro@ufh.ac.za (M.A.A.); 2 Department of Chemistry, University of Fort Hare, Private Bag X1314, Alice 5700, Eastern Cape, South Africa

**Keywords:** perovskite solar cells (PSCs), photovoltaic technologies, hole transport layer (HTL), power conversion efficiency (PCE)

## Abstract

Perovskite solar cells (PSCs) are regarded as extremely efficient and have significant potential for upcoming photovoltaic technologies due to their excellent optoelectronic properties. However, a few obstacles, which include the instability and high costs of production of lead-based PSCs, hinder their commercialization. In this study, the performance of a solar cell with a configuration of FTO/CdS/BaZrS_3_/HTL/Ir was optimized by varying the thickness of the perovskite layer, the hole transport layer, the temperature, the electron transport layer (ETL)’s defect density, the absorber defect density, the energy band, and the work function for back contact. Various hole transport layers (HTLs), including Cu_2_O, CuSCN, P3HT, and PEDOT:PSS, were assessed to select the best materials that would achieve high performance and stability in PSC devices. At optimal levels, PEDOT:PSS reached a maximum power conversion efficiency (PCE) of 18.50%, while P3HT, CuSCN, and Cu_2_O exhibited a PCE of 5.81, 10.73, and 9.80%, respectively. The high performance exhibited by PEDOT:PSS was attributed to better band alignment between the absorber and the PEDOT:PSS, and, thus, a low recombination of photogenerated charges. The other photovoltaic parameters for the best device were a short-circuit current density (J_sc_) of 23.46 mA cm^−2^, an open-circuit voltage (V_oc_) of 8.86 (V), and a fill factor (FF) of 8.90%. This study highlights the potential of chalcogenide-based PSCs as an efficient and stable alternative to traditional lead-based solar cells, with successful optimization paving the way for future research on eco-friendly materials and scalable production methods.

## 1. Introduction

The extensive use and reliance on fossil fuels as a major source of energy for power generation has led to implications for the environment and the global economy. Consequently, these fossil fuels result in the loss of resources, the deterioration of the environment, energy use, global warming, and climatic changes, thereby stimulating global economic growth [1,2,3]. Increasing population and economic growth have led to a search for sustainable energy sources, including renewable energy sources like solar, wind, and hydropower [4]. Solar energy is recognized as a low-carbon, high-power density, and sustainable source, making it a potential solution to the energy crisis [5,6]. Researchers are working on developing low-cost, reliable techniques for manipulating solar cells, highlighting the potential of photovoltaic systems as sustainable energy generation devices [7].

In emerging countries, solar photovoltaic (PV) technology is essential for low-income people because it converts solar energy into direct current, which lowers greenhouse gas emissions and dependency on fossil fuels [8]. As the third dominant renewable energy source, PV has seen a significant decrease in unit costs in the past five years [9]. Extensive photovoltaic research has contributed to the invention of different kinds of solar cells, such as perovskite solar cells (PSCs). PSCs are an affordable substitute for silicon- or lead-based solar cells, providing an optimal combination of productivity and the prospect of increasing the power conversion efficiency [10]. Recent years have witnessed tremendous growth in perovskite solar cells, with lead-based cells being the most efficient. However, these cells are potentially hazardous to the environment. Hence, this study was conducted to investigate lead-free perovskite solar cells with excellent light-to-electricity conversion efficiencies.

According to recent research and the NREL Chart, organic–inorganic hybrid halide perovskites demonstrate exceptional performance and quick advancement in the photovoltaic community, achieving 26.7% power conversion efficiency (PCE) in solar systems [11,12,13]. They are regarded as a significant scientific achievement in the development of absorber photovoltaic materials due to their intriguing optoelectronic features. However, Pb-based perovskites face instability due to organic cations and lead toxicity, while non-toxic Pb-free perovskites are being explored for their potential in solar photovoltaics. These materials, with their high device performance, can facilitate the economic viability of photovoltaic solar cells [14,15,16,17,18]. In 2009, Kojima et al. [19] created the first perovskite solar cell using methylammonium lead bromide (MAPbBr_3_) and methylammonium lead iodide (MAPbI_3_) as solid sensitizers. Lead halide perovskites have revolutionized PV materials, achieving a PCE of 3.8% in 2009 and 9.7% in 2012. Nevertheless, their toxicity in warmth, light, and moisture prevents them from being commercialized. Researchers are currently investigating lead-free perovskite materials to solve these environmental problems. In contrast to lead halide perovskites, chalcogenide perovskites provide better structural stability and non-toxic components, so they are now being investigated for solar applications [20]. BaZrS_3_ is a widely researched chalcogenide compound for optoelectronic applications. The study conducted by Chami et al. [21] reported chalcogenides as an alternative to lead methylammonium iodide, with a record-breaking PCE of 26%. Nonetheless, it has major ecological impacts resulting from the use of lead and its relative stability in natural environments.

**Figure 1:**
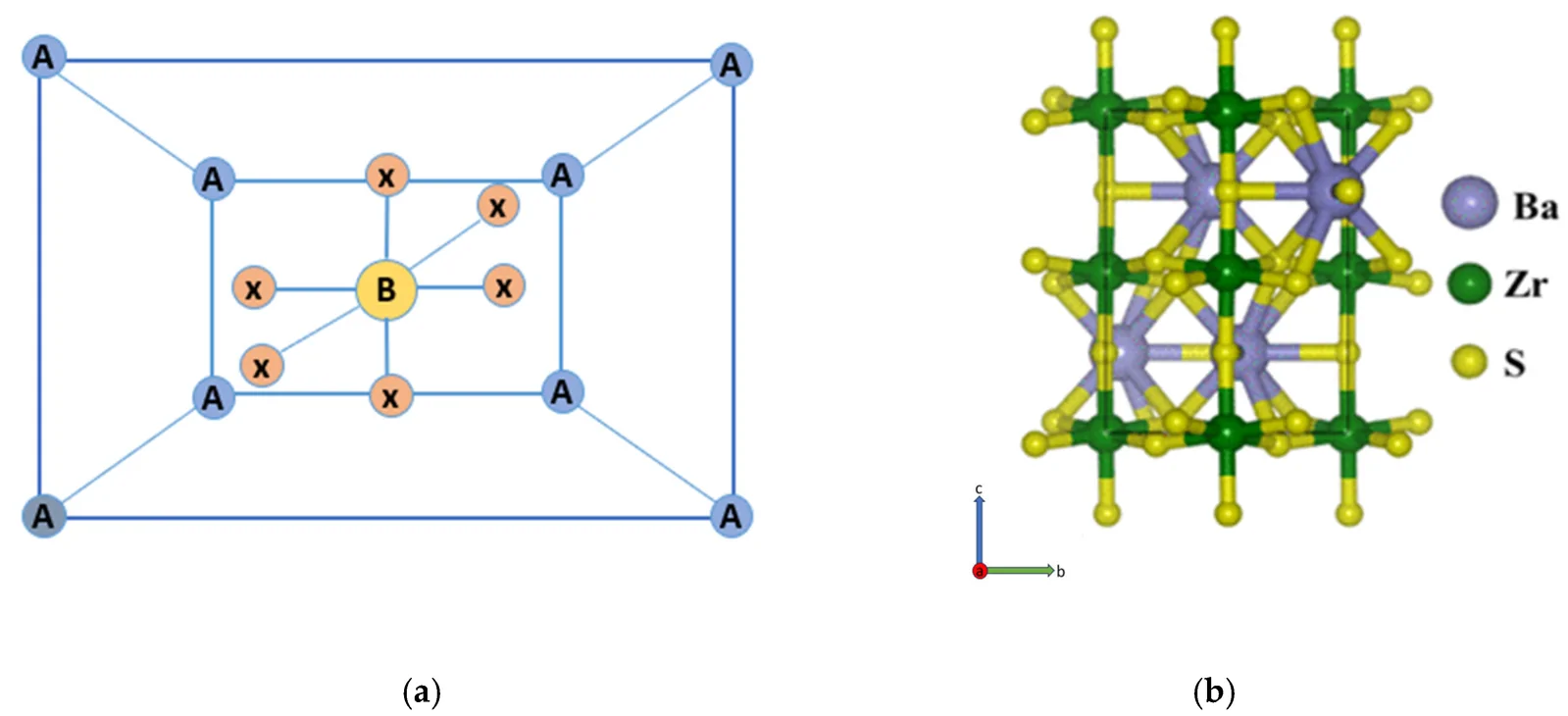
(**a**) ABX_3_ perovskite structure, where A represents the cation, B represents the divalent metal, and X represents the halide and (**b**) the crystal structure of BaZrS_3_ [27].

Perovskite possesses the chemical formula ABX_3_, derived from the first organic perovskite calcium titanate (CaTiO_3_), which is a naturally occurring crystal with a perfect cubic structure made up of corner-sharing BX_6_ octahedra [22,23]. A large cation size deforms the ABX_3_ structure of perovskite, affecting its behavior by adjusting ‘A’, ‘B’, and ‘X’ ion ratios (Figure 1). Lead-free solar cells currently perform poorly compared to lead-based designs; hence, this work was conducted to increase their power conversion efficiency and stability. Strategies include selecting appropriate HTL and ETL materials, minimizing shortcomings, and incorporating doping agents [24]. SCAPS 1D has been effectively utilized for simulating various perovskite solar cells (PSCs). For example, Umama et al. [25] employed this software to explore the performance of PSCs by combining Cs_3_Bi_2_I_9_, which has a high bandgap of (2.04 eV), CsSnGeI_3_, and CsSnI_3_, leading to an enhanced power conversion efficiency of 36.34%, revealing valuable insights into how to improve device efficiency. Their 2024 study underscores the versatility of SCAPS 1D in modeling a wide range of perovskite structures. This research highlighted the software’s ability to assess critical factors such as layer thickness, defect density, and temperature effects, which are essential for improving PSC performance and advancing solar technology. The study conducted by Zeng et al. investigated a novel approach to enhance the performance of wide-bandgap perovskite solar cells (PSCs) by utilizing work function-tuned PEDOT:PSS as the electron transport layer. They modified the traditional PEDOT:PSS by coating it with polyethyleneimine (PEI) to improve the electron transport efficiency within the solar cells and mitigate the degradation of power conversion efficiency (PCE). The findings indicated that the modified PEDOT:PSS significantly enhanced the stability of PSCs. Specifically, the degradation of solar cells was reduced from 29.7% to 15.6% after a testing period of 10 days. This improvement suggests that the modified material effectively contributed to maintaining the performance of the solar cells over time. The study indicated that modified PEDOT:PSS could be a viable candidate for commercial applications in PSC technology [26].

In this work, a solar cell capacitance simulator (SCAPS-1D) was used to demonstrate the numerical modeling and performance optimization of a chalcogenide-based perovskite solar cell whose primary architecture was FTO/CdS/BaZrS_3_/HTL/Ir. This was achieved by varying and optimizing the thickness of the perovskite layer, the hole transport layer, the temperature, the defect density of the absorber, the defect density of the ETL, the energy band, and the work function of the back contact. The most often used statistic for evaluating the efficacy of photovoltaic technology is the power conversion efficiency (PCE), which is the ratio of solar energy intake to electrical energy output. The efficiency consists of several system components, such as the short-circuit current density (J_sc_), the open-circuit voltage (V_oc_), and the fill factor (FF), which are reliant on fundamental material qualities and manufacturing defects [28]. Therefore, this study highlights the potential of chalcogenide-based PSCs as an efficient and stable alternative to traditional lead-based solar cells, with successful optimization paving the way for future research on eco-friendly materials and scalable production methods.

## 2. Device Configuration and Modeling

**Figure 2:**
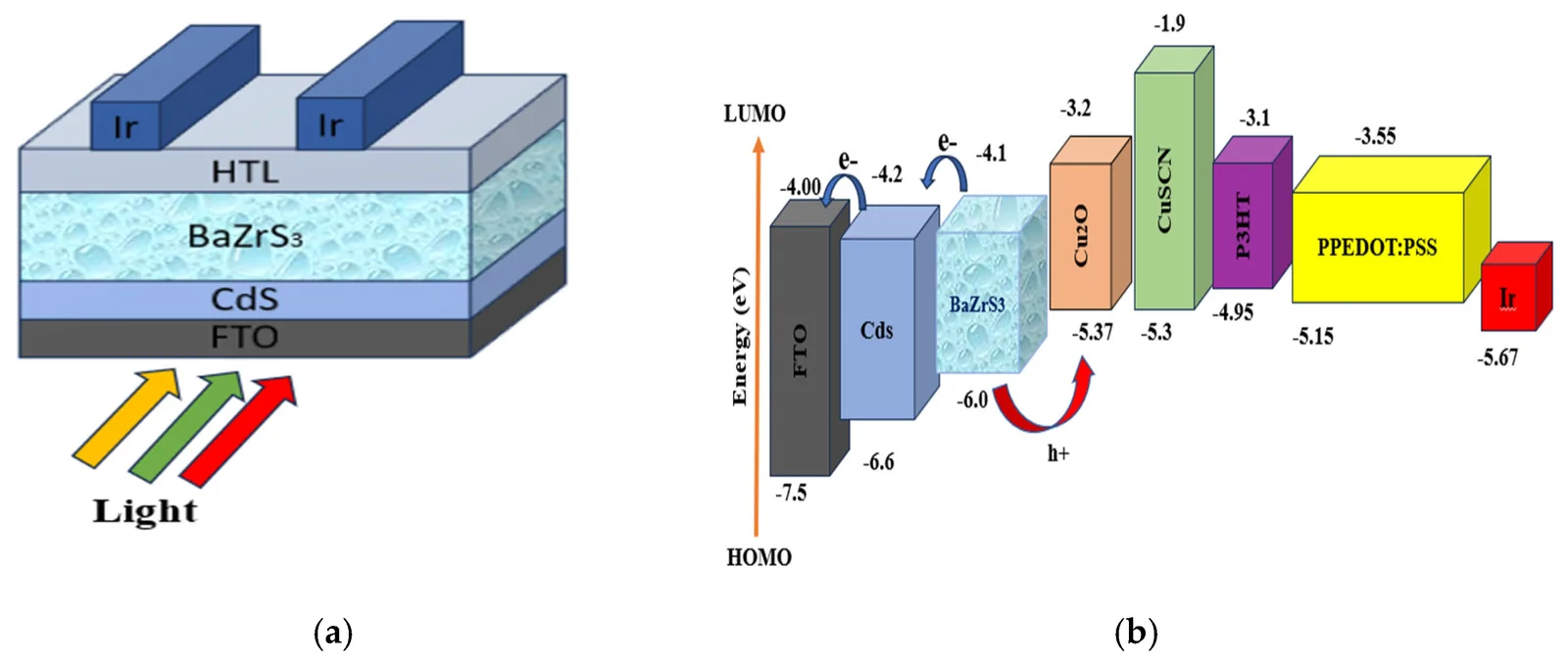
(**a**) Device structure of BaZr-S_3_-based perovskite and (**b**) energy band diagram.

This study utilized the SCAPS-1D software (version 3.3.10) [29], established by Professor Marc Burgelman and his fellow researchers at the University of Ghent, Belgium, in the computational modeling and optimization of our lead-free PSC. Figure 2 demonstrates the device schematics for the suggested design. It comprises BaZrS_3_ as the absorber layer, CdS as an electron transport layer (ETL), and several hole transport layers (HTLs) such as poly (3-hexylthiophene) (P3HT), copper (I) oxide (Cu_2_O), poly(3,4-ethylenedioxythiophene), and polystyrene sulfonate (PEDOT:PSS). The back metal contact was iridium (Ir), with a work function of 5.67 eV. The primary material characteristics were carefully chosen from published experimental and theoretical papers and tabulated in Table 1. The simulation was carried out on the n-i-p device with a configuration of FTO/CdS/BaZrS_3_/HTL/Ir (Figure 2a), and the corresponding band alignment of the selected materials is presented in Figure 2b. SCAPS-1D is a flexible simulation tool that can handle multi-layered systems such as heterojunctions and thin-film solar cells. It supports parameter modification, charge transport modeling, energy band diagrams, interface and defect analysis, and performance evaluation. However, it includes drawbacks such as a one-dimensional model, a simplified optical model, a small material library, non-idealities, and a lack of time-dependent simulations [30,31,32].

## 3. Results and Discussions

**Figure 3:**
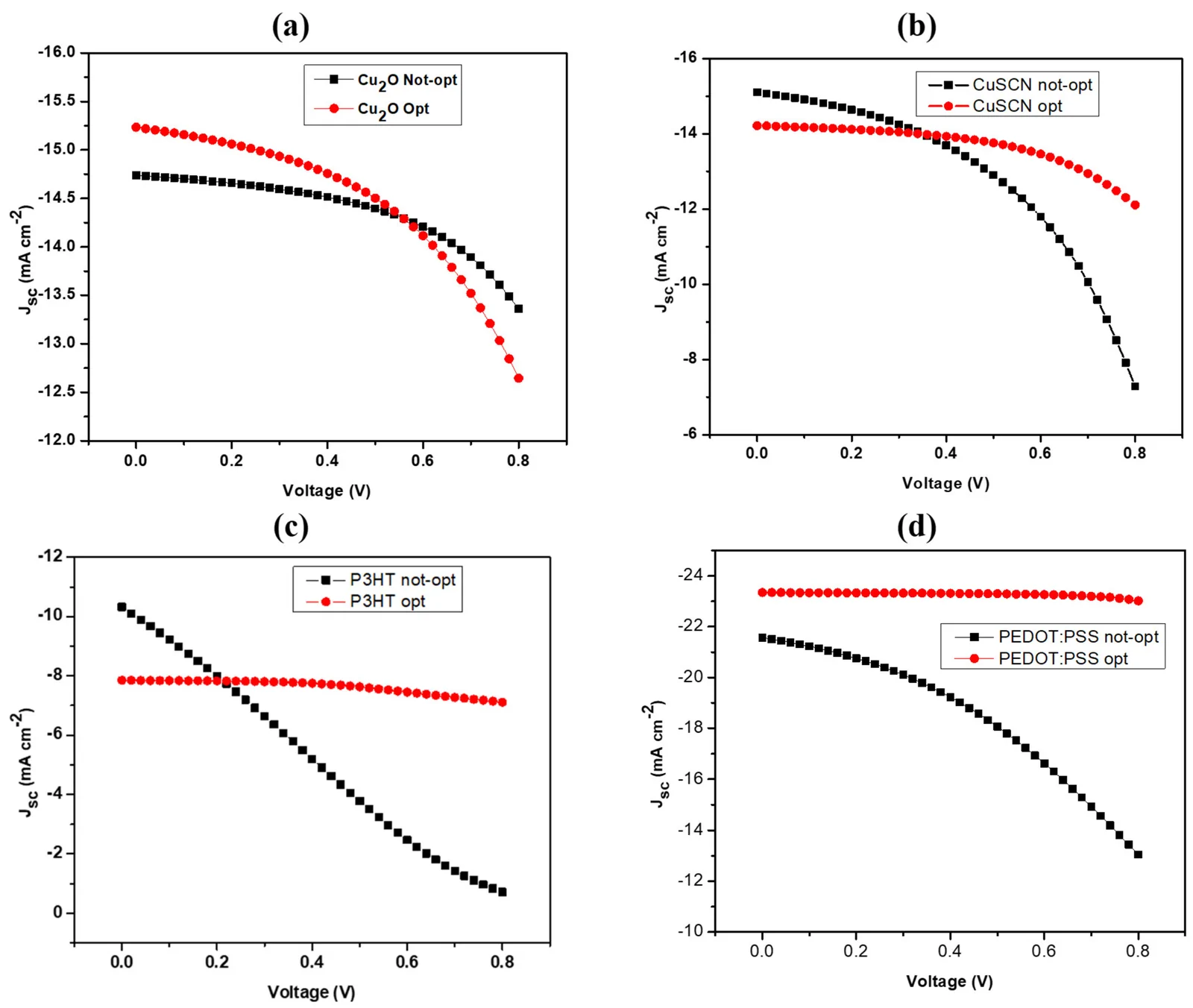
J--V curves for optimized and non-optimized devices with different HTLs: (**a**) Cu_2_O, (**b**) CuSCN, (**c**) P3HT, and (**d**) PEDOT:PSS.

The numerical simulation of a lead-free chalcogenide-based perovskite cell was performed using the SCAPS-1D software. Various materials such as Cu_2_O, CuSCN, P3HT, and PEDOT:PSS were selected and employed as the HTL to examine their impact on the PV performance of the primary n-i-p device. The simulation results were obtained by observing photovoltaic features like the J_sc_, V_oc_, FF, and PCE, using parameters from Table 1. Thickness optimization was carried out by varying the thickness of one layer while keeping the others fixed. For example, the thickness for Cu_2_O was varied from 1.00 µm to 2.00 µm, while FTO, Cds, and BaZrS_3_ were fixed at 0.10, 1.60, and 0.40 µm. Optimal performance was obtained at 1.5 µm. Then, for Cds, the thickness was varied from 1.00 to 2.00 µm, and the optimal thickness was obtained at 1.6 µm, while other materials were fixed starting from the first values. After the optimization of the thickness of the ETL, the absorber (BaZrS_3_) was varied from 0.10 to 1.00 µm, and the best thickness was achieved at 0.4 µm. Finally, the thicknesses of Cds, Cu_2_O, and BaZrS_3_ were fixed at optimized values, and the FTO thickness was varied from 0.01 to 0.50 µm. The highest performance was achieved with a 0.10 µm thicker FTO material. This procedure was repeated for other devices with CuSCN, P3HT, and PEDOT:PSS as the HTL materials, and each layer was explored separately until a maximum PCE was obtained. Figure 3 shows the current density versus the voltage (J-V) for modeled devices before and after thickness optimization. In this study, we investigated the photovoltaic properties of various kinds of devices with different HTL materials, such as P3HT, Cu_2_O, and PEDOT:PSS. The J_sc_ curves of all non-optimized devices decreased with increasing voltage, while, for optimized devices, they decreased for (a) and (b), with (c) and (d) remaining constant at 8.00 and 23.46 mA cm^2^, respectively. This showed that optimized devices performed better than non-optimized devices and that PEDOT:PSS has more efficient light absorption and charge [39].

### 3.1. Effect of Different Hole Transport Layer Materials on the Photovoltaic Performance

**Figure 4:**
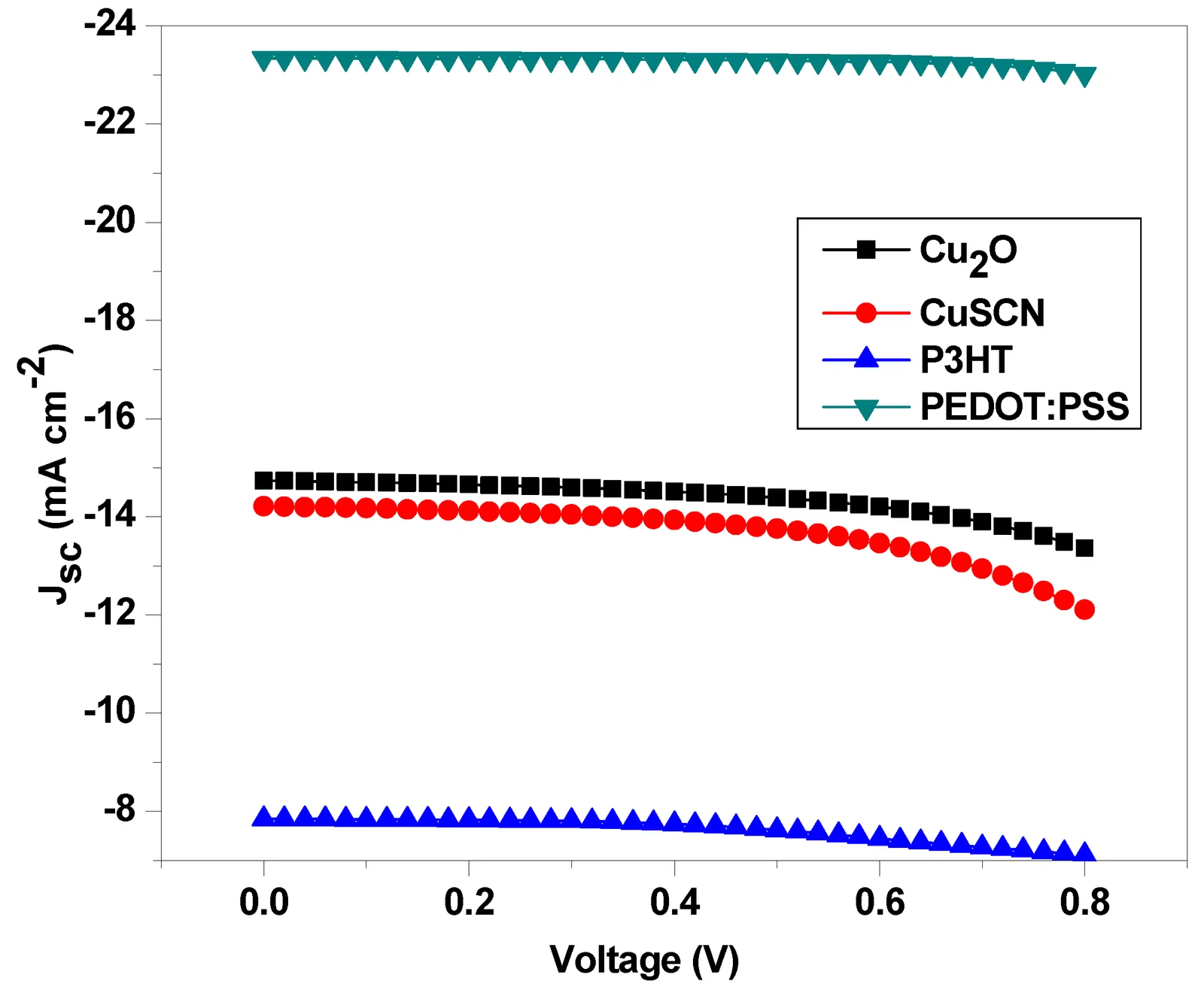
Simulated J-V curve of BaZrS_3_-based solar cells with different HTL materials.

Figure 4 depicts the optimized performance parameters acquired from simulations with various HTLs, while Table 2 summarizes the retrieved photovoltaic output values for all modeled devices. It can be observed that PEDOT:PSS achieved a much improved value of the short circuit current density (J_sc_) of 23.46 mA cm^−2^ and, thus, the highest PCE of 18.50%. The best performance by PEDOT:PSS could be attributed to better charge transfer characteristics, due to better band alignment. Meanwhile, Cu_2_O and CuSCN offered a much closer value of J_sc_ due to the compatible mobility of the carriers. P3HT had relatively lower PCE and V_oc_ than the other devices. The low performance exhibited by the P3HT device was attributed to the severe recombination and low hole mobility of about 1.0 × 10^−3^ cm^2^ V^−1^ s^−1^ (please see Table 1), which might have impacted the charge collection mechanism.

### 3.2. Quantum Efficiency Analysis

**Figure 5:**
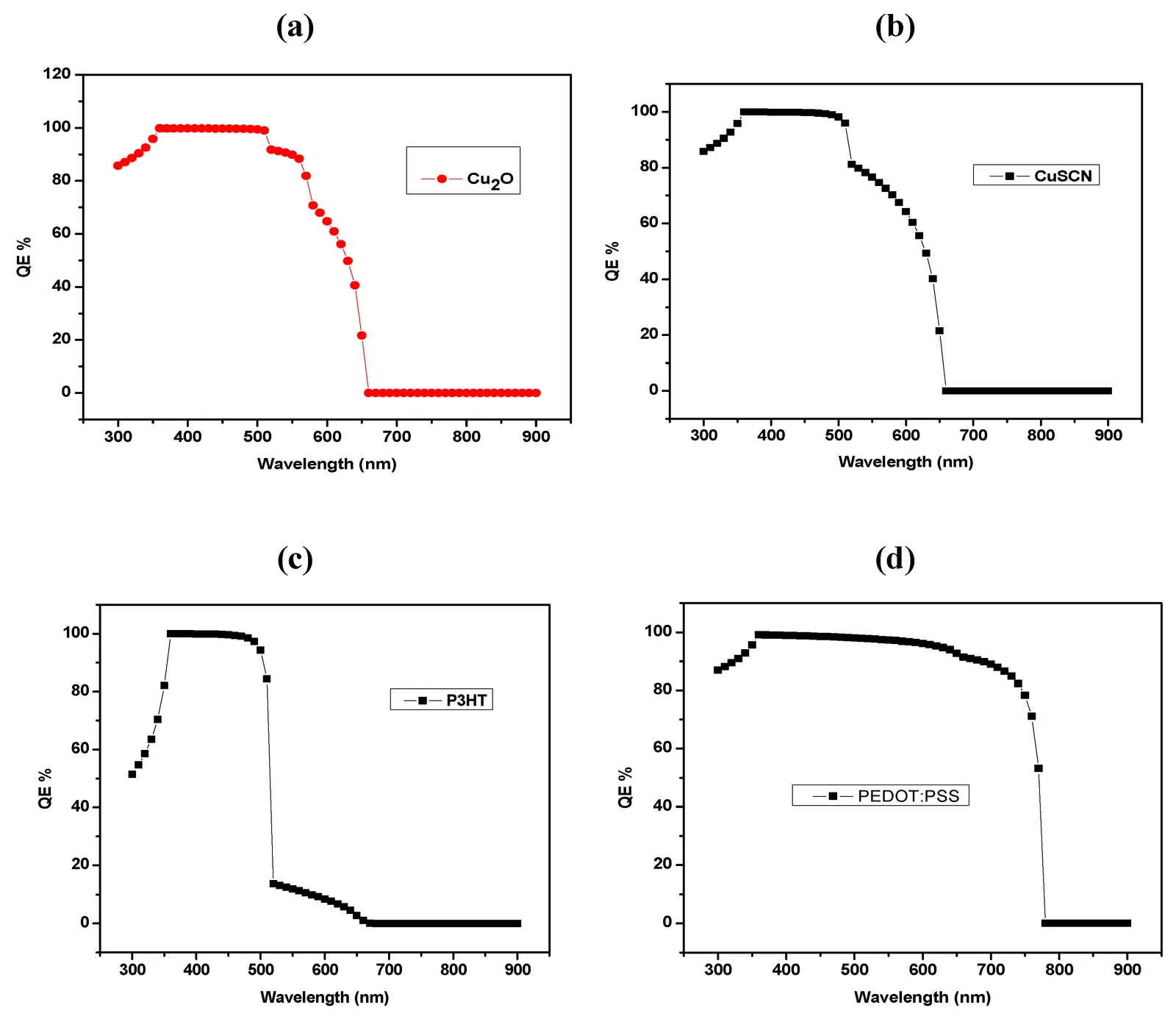
Relationship between quantum efficiency (QE) and wavelength (nm) for (**a**) Cu_2_O-, (**b**) CuSCN-, (**c**) P3HT-, and (**d**) PEDOT:PSS-based devices.

The QE values of all the devices showed a rise from 300 to 350 nm, with the graphs in Figure 5a,b,d having their maximum of 80–100% efficiency between 300 and 350 nm, while, in Figure 5c, they reached a maximum between 300 and 500 nm. Conversely, from 550 to 650 nm wavelength, the QE for the devices in Figure 5a and Figure 5b dropped from 100% to 0%, while, for the devices in Figure 5c and Figure 5d, it fell sharply at 500 nm and 780 nm, respectively. This implies that the devices could absorb ultraviolet and visible light only for that portion of the wavelength. Contrary, the PEDOT:PSS-based device exhibited unique QE. This implies that the simulated devices could only absorb light within the visible range of the electromagnetic spectrum and not the infrared radiation.

### 3.3. Impact of n-Type Doping Density (N_D_) of the CdS-Based ETL

**Figure 6:**
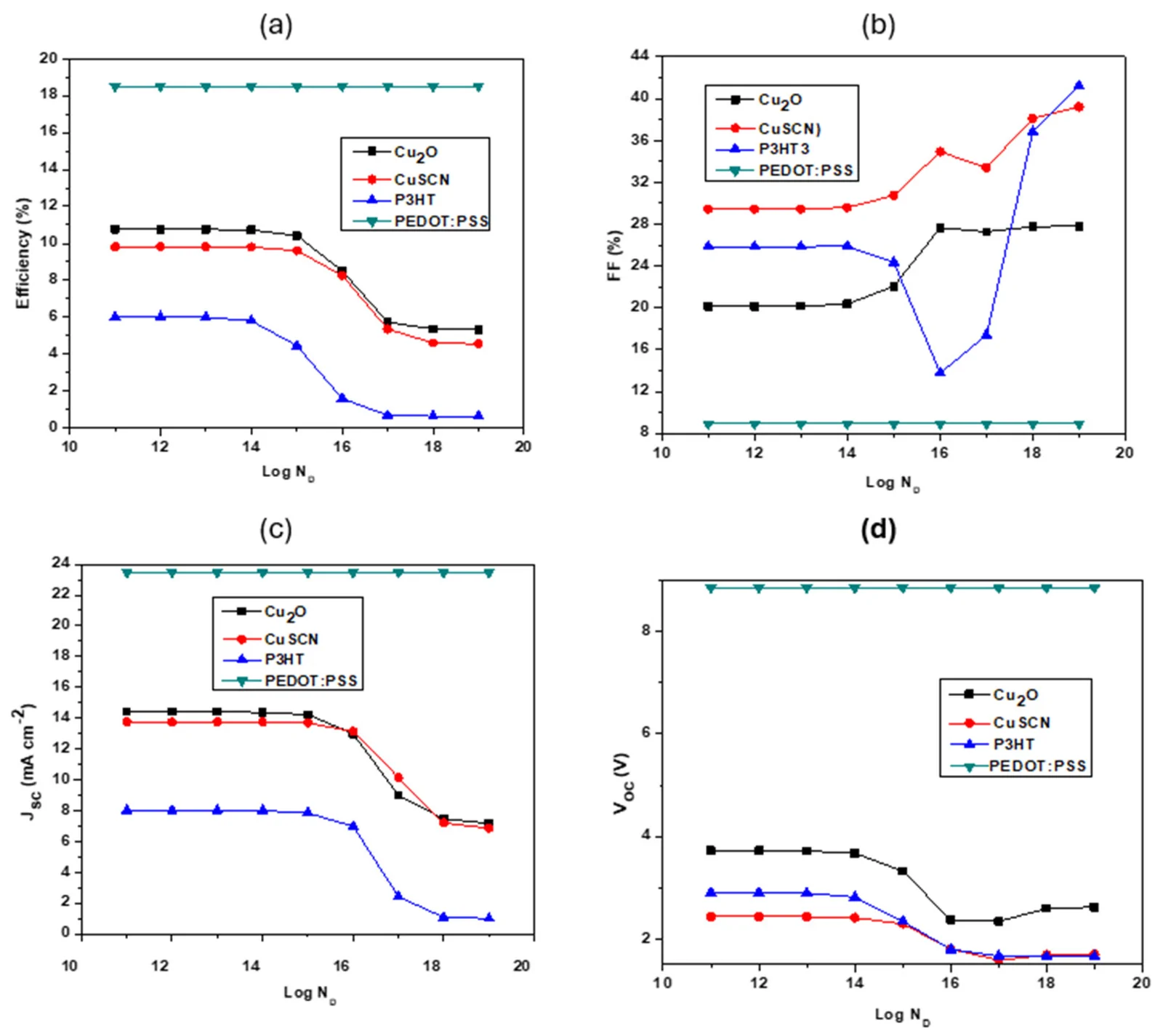
Effect of n-type doping density for CdS ETL on PSCs. (**a**) PCE, (**b**) FF, (**c**) J_sc_ and (**d**) V_oc_

Doping enhances the performance of solar cells by altering the optoelectronic qualities of the HTL, ETL, and absorber components. Figure 6 illustrates that, as the doping density increases within the CdS-based ETL of the devices, J_sc_ remains constant between 1.00 × 10^11^ cm^−3^ and 1.00 × 10^14^ cm^−3^ and declines from 1.00 × 10^15^ cm^−3^ to 1.00 × 10^19^ cm^−3^ for the devices in Figure 6a, Figure 6c, and Figure 6d, except for the device in Figure 6b, for which it increases at 1.00 × 10^15^ cm^−3^. However, the device based on PEDOT:PSS was constant throughout, with V_oc_, J_sc_, and PCE values outperforming the others but the lowest constant FF values. The best performance could be attributed to the better charge transfer characteristics within the device. Nonetheless, there was no significant impact of n-type doping on the PEDOT:PSS-based device, and this was attributed to the fact that, probably, the absorber and the HTL were much more involved in light absorption and fast charge transfer than the CDS ETL; thus, its n-doping did not have an impact on the photovoltaic parameters. The PEDOT:PSS had an optimal doping density with a maximum J_sc_ of 23.46 mA cm^−2^. The decrease in performance could be attributed to the accumulation of extra electrons, which caused slow conductivity, impacting the cell performance, and the extra concentration may have induced cell damage and failure [40]. PEDOT:PSS surpassed other HTLs in terms of V_oc_ and PCE values, but its fill factor was limited due to a balance between hole mobility and charge recombination, with excessive doping reducing efficiency and inadequate doping increasing conductivity. In comparison to PEDOT:PSS, P3HT exhibited inferior performance indicators and a lower fill factor. This was owing to significant recombination losses and reduced hole mobility, which impeded effective charge collection and efficiency. P3HT’s fill factor did not considerably improve with increasing voltage, indicating that it struggled to sustain effective charge extraction at higher working voltages. This draws attention to the drawbacks of P3HT as an HTL with regard to charge transmission and overall device functionality. Furthermore, increasing ETL doping density raises the interfacial electric field. More importantly, a higher ETL doping density enhances the interfacial electric field within the absorber layer and ETL, thereby improving the ability to extract holes and electrons from it by lowering the rate of recombination and separating exciton [34].

### 3.4. Impact of Defect Density (N_t_) on Performance

**Figure 7:**
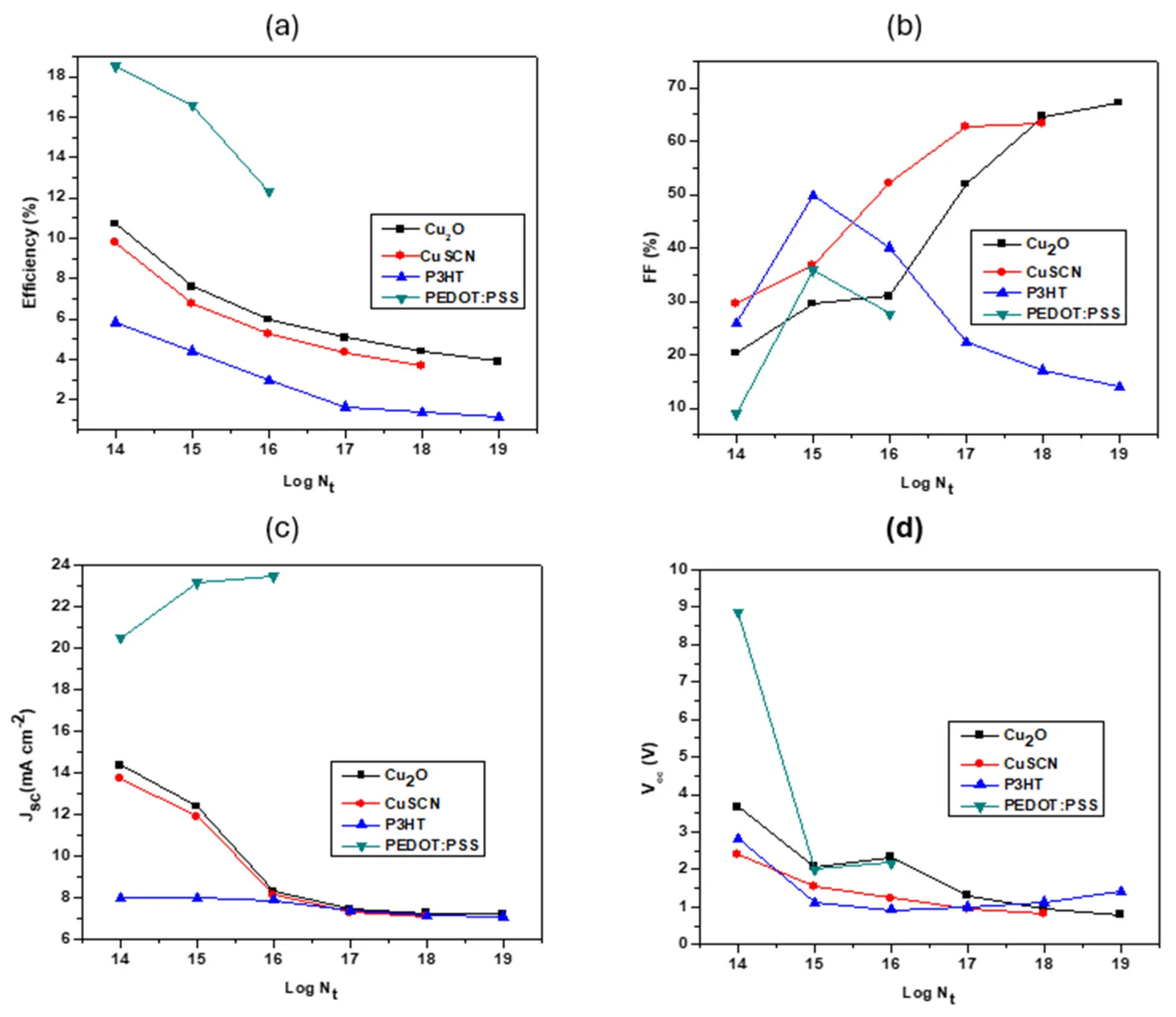
Effect of density of defect for the absorber on PSCs. (**a**) PCE, (**b**) FF, (**c**) J_sc_ and (**d**) V_oc_

Photogenerated carriers and recombination processes have a significant influence on the performance of PSCs. Low-quality perovskite layers cause higher recombination rates, leading to carrier loss and shorter diffusion lengths and lifetimes. Figure 7 depicts the trend in the density of defect on fuel cell performance. To determine the optimal defect density for simulated cells, the density varied from 1.00 × 10^14^ to 1.00 × 10^19^ cm^−3^, and the devices were not functioning below 1.00 × 10^14^ cm^−3^. The PCE, J_sc_, and V_oc_ of the devices dropped dramatically as the density of defects increased due to the higher rate of charge recombination. Meanwhile, the FF for Cu_2_O and CuSCN increased with an increase in the absorbing width. For the device of PEDOT:PSS, it was not functioning beyond 1.00 × 10^16^ cm^−3^. This shortened the diffusion route length of charge carriers and their lifetimes. The decrease in the performance of the cells was due to the traps that captured charge carriers, preventing them from taking part in the current flow of the solar cells, and a higher defect density promoted electron and hole recombination, which hindered their efficient current generation and lowered the V_oc_. However, tuning the density of defects can be very expensive at the industrial level, and the choice of production method can significantly alter the microstructural properties of materials, thereby influencing their electronic characteristics. Also, the material structures have a substantial influence on industrial performance, with the density of defect and the distribution influencing the mechanical quality and dependability. Furthermore, a higher defect density might result in increased expenses and quality control. Material interaction influences contact formation, which impacts wear resistance and electrical conductivity in applications such as electrical contacts and bearings. Understanding surface roughness and material hardness can boost performance in high-stress circumstances. However, errors and improved production procedures sometimes come with increased expenses.

### 3.5. Effect of Temperature on Cell Performance

**Figure 8:**
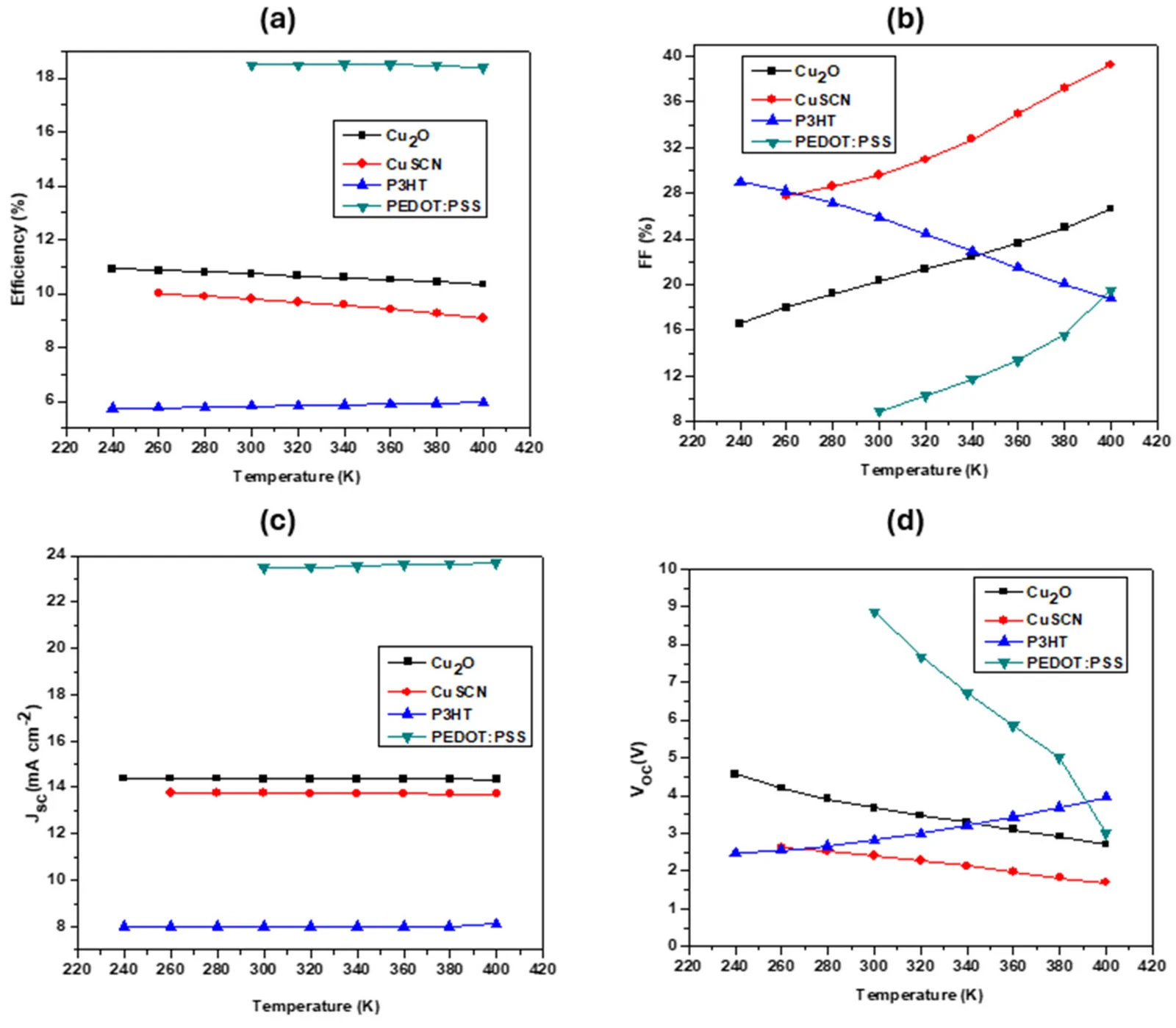
Effect of temperature variation on photovoltaic cells. (**a**) PCE, (**b**) FF, (**c**) J_sc_ and (**d**) V_oc_

The operating temperature has a considerable influence on the performance of solar cells in real-world settings. Solar cells are subjected to shifting weather conditions, and the stability of the device may be compromised if its temperature is twice that of the surrounding environment. A solar cell can operate at temperatures as high as 300 K, although this is the standard operating temperature. Thermal stability was assessed by varying the temperature from 240 K to 400 K. Figure 8 shows the influence of temperatures on the photovoltaic properties of the systems using a typical CdS ETL, the BaZrS_3_ absorber, and several HTL materials. The increase in temperature increased the FF of the devices having Cu_2_O, CuSCN, and PEDOT:PSS HTLs and the V_oc_ of P3HT, while the PCE and J_sc_ remained stable for all the devices. However, the device using P3HT had increased values, while the FF declined. The stability indicated that there was no photovoltaic performance observed. Furthermore, the devices comprising PEDOT:PSS did not function at temperatures below 300 K, yet operated admirably in terms of V_oc_ and FF, indicating that these device might have been unstable. Meanwhile, J_sc_ was marginally shifting for all HTLs. However, the cell with P3HT proved to be stable, as indicated by the virtually consistent PCE over the 260 to 400 K temperature range. Notwithstanding its lowest PCE, the P3HT cell demonstrated stability as seen by its reasonably constant PCE over the 260 to 400 K temperature range. This indicates that these devices are sensitive and may fail at high temperatures.

### 3.6. Optimization of Back Contact Work Function

The performance of solar cells was assessed using various metal back contacts. The metals examined were Ir (5.67 eV), Pt (5.93 eV), Ag (4.74 eV), Au (5.47 eV), and Cu (4.36 eV). The researchers discovered that the metal used had a significant influence on the work function of a back contact material (HTL). According to Table 3, Pt and Ir were the best back contacts for all model cells due to their promising electrical properties. Pt (5.93 eV), with its low resistance, easily pulled charges from the perovskite layers, lowering energy losses and improving device performance by allowing for robust electrical contacts with various solar cells [41,42]. It demonstrated equivalent or comparable work function values to Ir (5.67 eV) for each HTL, indicating similar electrical interactions, whilst Au (5.47 eV) was a more affordable alternative. Ag (4.74 eV) and Cu (4.36 eV) exhibited lower work function values, with Ag showing the most severe reduction across all HTLs. This study found that PEDOT:PSS (18.50%) performed best for high-work activities, whereas P3HT (5.81%) was preferred for steady work functions. High-work function metals, in particular, enhanced cell performance by establishing a high barrier that prevented electrons from moving from the hole transport material to the metal contact. The results show that Pt, Ir, and Au may be employed as back contacts in Cu_2_O, CuSCN, and PEDOT:PSS hole transport devices. However, additional metal back qualities like cost, toxicity, and stability should be taken into account while building a device configuration.

### 3.7. Energy Band Diagram

**Figure 9:**
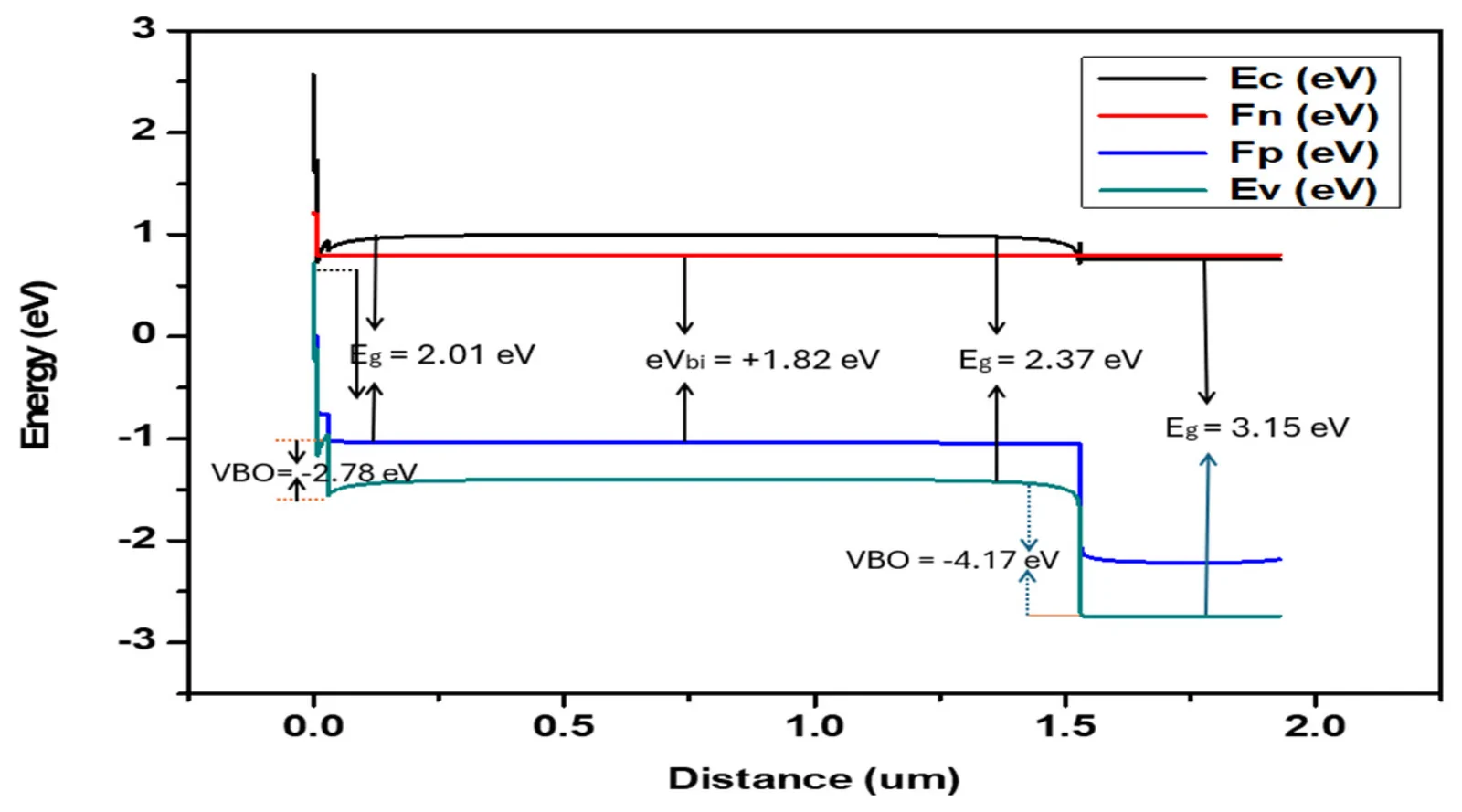
Energy band diagram of Cu_2_O device.

Understanding the performance of a solar cell requires knowledge of the energy band diagram. This defines the energy levels that electrons can occupy within the cell, emphasizing the distinction between the valence band and the conduction bands. When a cell absorbs sunlight, electrons are stimulated from the valence band to the conduction band, producing an electrical current. Figure 9 displays the PSC energy band diagram from the SCAPS-1D program, using Cu_2_O as the hole transport layer. The BaZrS_3_-based PSC has a lower energy barrier (almost +0.8 eV) between the EC of ETL. Cliffs may be caused by the non-uniformity of the band diagram. This graphic may also be used to assess the efficiency of a solar cell, noting losses caused by recombination or other reasons at the interfaces. The presence of a cliff may have led to the suppressed recombination of the photogenerated charges and, thus, better performance.

### 3.8. Dark/Indoor Simulation Consideration

**Figure 10:**
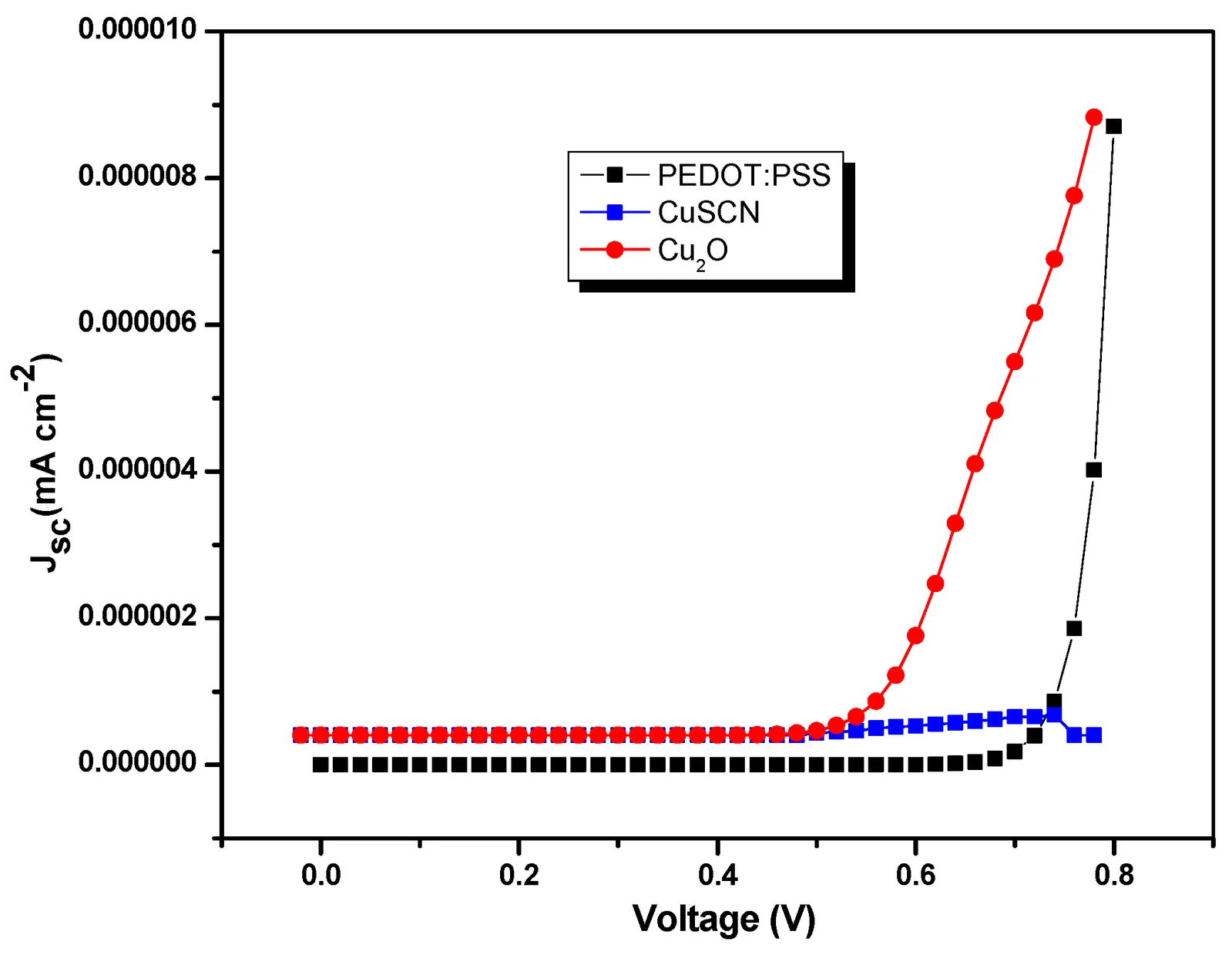
Dark illumination spectrum for the current density–voltage (J–V) curve.

Indoor lighting conditions differ significantly from natural sunshine, since conventional indoor lights such as LEDs and fluorescents have a lower intensity and differing spectrum distributions. This variance in illumination can result in consistent performance measures, which could be attributed to the precise light spectrum employed during simulations or measurements [43,44,45]. By examining the current density–voltage (J-V) characteristics of perovskite solar cells under dark conditions in Figure 10, we compared various hole transport materials: PEDOT:PSS, CuSCN, and Cu₂O. Unfortunately, P3HT failed to operate under these dark conditions, likely due to several technical challenges related to its material properties, device structure, and electrical characteristics, such as inadequate transport layer properties, current leakage, recombination losses, etc.

In contrast, PEDOT:PSS exhibited essentially flat J-V characteristics between 0.0 and 0.7 V, indicating that it generated relatively little current. This implies that it may not be the ideal option for interior lighting applications unless further tuned. Meanwhile, CuSCN produces some current, but its output is relatively modest compared to Cu₂O. This indicates moderate performance and may require extra modifications for optimal use in indoor conditions. Lastly, Cu₂O has an improved performance and generates much larger currents, especially at higher voltages (0.5 to 0.8 V), making it ideal for interior lighting applications. Therefore, the devices perform well on the light illumination spectrum.

## 4. Conclusions

The current study effectively developed and numerically investigated the environmentally friendly chalcogenide BaZrS_3_-based PSCs utilizing the SCAPS-1D software. Several proposed HTL materials, including inorganic Cu_2_O, CuSCN, and organic P3HT and PEDOT:PSS, were inserted and tested. The best-performing device used PEDOT:PSS as the HTL. The optimal efficiency achieved by the best device was a PCE of 18.50%, and the total defect density was kept at 1.00 × 10^14^ cm^−3^ and was applied to all the devices. The P3HT-based device achieved the lowest performance with a PCE of 5.81% which was attributed to the lower electron mobility and high recombination rate of photogenerated charges. Generally, all the devices showed appreciable photovoltaic performances even in the dark, except for the P3HT-based device, which indicates that these devices can be used in indoor environments. An important contribution of the informative simulated results in this work is to provide insights for the experimental understanding of a sustainable and viable perovskite solar cell (PSC). Thus, the results obtained may be useful in the correct design of high-performance BaZrS_3_ PSCs and the reasonable selection of the necessary hole transport material in the future.

## Figures and Tables

**Table 1:** Parameters of the input layers used to simulate the designed device.

Parameters	FTO [24]	CdS [33,34]	BaZrS_3_ [2]	Cu_2_O [35,36]	CuSCN [37]	P3HT [37]	PEDOT:PSS [38]
Bandgap, Eg (eV)	3.50	2.40	1.90	2.17	3.40	1.85	1.60
Electron affinity, χ (eV)	4.00	4.20	4.10	3.20	1.90	3.10	3.55
Dielectric Permittivity (relative) e_r_	9.00	10.00	9.60	7.11	10.00	3.40	2.58
CB density of states, Nc (cm^–3^)	2.20 × 10^18^	2.20 × 10^18^	2.20 × 10^18^	2.02 × 10^17^	1.70 × 10^19^	1.00× 10^22^	2.10 × 10^21^
VB density of states, Nv (cm^–3^)	1.80 × 10^19^	1.80 × 10^19^	1.80 × 10^19^	1.10 × 10^19^	2.50 × 102^21^	1.00 × 10^22^	2.00 × 10^21^
Electron mobility, μe (cm^2^ V^−1^ s^−1^)	20.00	350.00	1.70 × 10^−2^	2.00 × 10^2^	10.00 × 10^−4^	1.00 × 10^−4^	1.00
Hole mobility, μh (cm^2^ V^−1^ s^−1^)	10.00	50.00	5.90 × 10^−2^	8.00 × 10^1^	1.00 × 10^−1^	1.00 × 10^−3^	20.00
Density n-type doping, *N*_D_ (cm^–3^)	1.00 × 10^19^	1.00 × 10^15^	1.00 × 10^12^	0.00	0.00	0.00	0.00
Density p-type doping, *N*_A_ (cm^–3^)	0.00	0.00	1.00 × 10^12^	1.00 × 10^18^	1.00 × 10^18^	3.17 × 10^13^	3.00 × 10^20^
Defect density, N_t,_ (cm^−3^)	0.00	1.00 × 10^14^	1.00 × 10^15^	1.00 × 10^14^	1.00 × 10^14^	1.00 × 10^14^	1.00 × 10^14^
Optical absorption constant A, (cm^−1^ eV^1/2^)	5.345 × 10^4^	5.345 × 10^4^	5.345 × 10^4^	5.345 × 10^4^	5.345 × 10^4^	5.345 × 10^4^	5.345 × 10^4^
Optical absorption constant B, (eV^1/2^)	1.871 × 10^−12^	1.871 × 10^−12^	1.871 × 10^−12^	1.871 × 10^−12^	1.871 × 10^−12^	1.871 × 10^−12^	1.871 × 10^−12^

**Table 2:** Photovoltaic output parameters of the modeled devices with different HTL materials.

Parameter	Cu_2_O	CuSCN	P3HT	PEDOT:PSS
**PCE (%)**	10.09	9.80	5.81	18.50
**FF (%)**	23.23	29.58	25.88	8.90
**V_oc_ (V)**	3.02	13.74	2.81	8.86
**J_sc_ (mA cm^−2^)**	14.37	2.41	8.00	23.46

**Table 3:** Percentage PCE values for devices utilizing different HTLs and metal back contacts.

HTL	Ir	Au	Ag	Cu	Pt
**Cu_2_O**	10.73	10.72	8.90	10.59	10.73
**CuSCN**	9.80	9.77	4.53	6.64	9.80
**P3HT**	5.81	5.81	5.65	5.78	5.81
**PEDOT:PSS**	18.50	18.22	16.65	16.66	18.50

## Data Availability

The original contributions presented in this study are included in the article. Further inquiries can be directed to the corresponding author.
